# Physiological and metabolic characteristics of novel double‐mutant female mice with targeted disruption of both growth hormone‐releasing hormone and growth hormone receptor

**DOI:** 10.1111/acel.13339

**Published:** 2021-03-23

**Authors:** Mert Icyuz, Fang Zhang, Michael P. Fitch, Matthew R. Joyner, Anil K. Challa, Liou Y. Sun

**Affiliations:** ^1^ Department of Biology University of Alabama at Birmingham Birmingham Alabama USA

**Keywords:** CRISPR, GHR, GHRH, indirect calorimetry, insulin sensitivity, metabolism

## Abstract

Mice with disruptions of growth hormone‐releasing hormone (GHRH) or growth hormone receptor (GHR) exhibit similar phenotypes of prolonged lifespan and delayed age‐related diseases. However, these two models respond differently to calorie restriction indicating that they might carry different and/or independent mechanisms for improved longevity and healthspan. In order to elucidate these mechanisms, we generated GHRH and GHR double‐knockout mice (D‐KO). In the present study, we focused specifically on the characteristics of female D‐KO mice. The D‐KO mice have reduced body weight and enhanced insulin sensitivity compared to wild‐type (WT) controls. Growth retardation in D‐KO mice is accompanied by decreased GH expression in pituitary, decreased circulating IGF‐1, increased high‐molecular‐weight (HMW) adiponectin, and leptin hormones compared to WT controls. Generalized linear model‐based regression analysis, which controls for body weight differences between D‐KO and WT groups, shows that D‐KO mice have decreased lean mass, bone mineral density, and bone mineral content, but increased adiposity. Indirect calorimetry markers including oxygen consumption, carbon dioxide production, and energy expenditure were significantly lower in D‐KO mice relative to the controls. In comparison with WT mice, the D‐KO mice displayed reduced respiratory exchange ratio (RER) values only during the light cycle, suggesting a circadian‐related metabolic shift toward fat utilization. Interestingly, to date survival data suggest extended lifespan in D‐KO female mice.

## Graphical abstract

In comparison with WT mice, the D‐KO mice displayed reduced respiratory exchange ratio (RER) values only during the light cycle, suggesting a circadian‐related metabolic shift towards fat utilization.

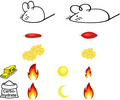

## 1 INTRODUCTION

Growth hormone (GH) is a peptide hormone, mainly synthesized by specialized cells called somatotrophs located in the anterior lobe of the pituitary gland (Bartke et al., 2013; Chandrashekar et al., 1988). Growth hormone‐releasing hormone (GHRH) and somatostatin, also known as GH‐inhibiting hormone, are both produced in the hypothalamus and are the primary regulators of GH secretion (Brar et al., 1989; R. G. Smith, 2005; Suhr et al., 1989). The GH receptor (GHR) protein and GH‐binding protein (GHBP) are encoded by the GHR/GHBP gene and are generated by alternative splicing in mice (W. C. Smith et al., 1988). The impact of GH signaling on dramatic extension of longevity was initially discovered in Snell dwarf mice, which were homozygous for a recessive mutation in the pituitary factor 1 (Pit‐1) (Camper et al., 1990; Li et al., 1990; Mangalam et al., 1989; Simmons et al., 1990). Several decades later, Ames dwarf mice, which had a recessive mutation in the Prophet of Pit‐1 (Prop‐1) gene, were found to have a very similar long‐lived phenotype (Andersen et al., 1995; H. M. Brown‐Borg et al., 1996). Both Pit‐1 and Prop‐1 genes are expressed in the anterior pituitary and regulate the development of cells into somatotrophs, thyrotrophs, and lactotrophs, which synthesize GH, thyroid‐stimulating hormone, and prolactin, respectively (Bartke & Brown‐Borg, 2004; Sornson et al., 1996).

To delineate the role GH signaling on lifespan, Zhou et. al 1997 disrupted the growth hormone receptor/binding protein gene generating the long‐lived mouse line with GH resistance, mimicking Laron dwarfism observed in humans, who have reduced risk of cancer and type 2 diabetes (Coschigano et al., 2003; Flurkey et al., 2001; Zhou et al., 1997). The dramatic effect of GH signaling was also supported with the long‐lived GH deficiency model, which was generated by knocking out GHRH gene in mice (Aguiar‐Oliveira et al., 2010; Sun et al., 2013).

GH signaling has demonstrated the strongest impact on longevity independent of genetic background and diet in mice (Bonkowski et al., 2006; Coschigano et al., 2003; Sun et al., 2013). These animals share numerous characteristics relevant to their prolonged lifespan, including decreases in the rates of growth, sexual maturation, adult body size, body temperature, circulating insulin, glucose levels and increases in insulin sensitivity, adiposity, expression of genes involved in xenobiotic detoxification and stress resistance (Amador‐Noguez et al., 2007; Borg et al., 1995; H. Brown‐Borg et al., 2001; Coschigano et al., 2003; Flurkey et al., 2001; Kennedy et al., 2003; Liu et al., 2004; Masternak et al., 2009; Parsons et al., 1995; Salmon et al., 2005; Sun et al., 2013; Zhou et al., 1997).

The GH‐resistant and GH‐deficient mice respond differently to calorie restriction (CR). We and others have shown previously that CR significantly extends longevity in WT animals from these strains, and it causes a further extension of longevity in GH‐deficient mice (Bartke et al., 2001; Sun et al., 2013), but fails to affect longevity of GHR‐KO males and has only a small effect on maximal (but not median or average) longevity of GHR‐KO females (Bonkowski et al., 2006). These observations indicate that GH resistance and GH deficiency might carry out different and/or independent mechanisms for improved longevity and healthspan. In order to elucidate the exact mechanism, we have generated GHRH and GHR double‐knockout mice (D‐KO).

In this study, we targeted two major genes of the GH signaling pathway: growth hormone‐releasing hormone and growth hormone receptor/growth hormone‐binding protein using the CRISPR/Cas9 technology. The simultaneous disruption of GHRH and GHR genes resulted in dramatically decreased body weight, higher insulin sensitivity, and glucose intolerance. The double‐knockout mice have significantly reduced bone mineral density (BMD), bone mineral content (BMC), and lean mass. However, the double‐knockout mice have significantly increased fat mass compared to littermate controls. We performed extensive indirect calorimetry to gain insight into the respiratory and metabolic features of the novel mutant line. We observed significantly reduced oxygen consumption, carbon dioxide production, and energy expenditure during both light and dark cycles. Respiration exchange ratio was significantly lower during the light cycle, but not dark cycle.

Our study presents a novel GH signaling disrupted model, which combines GH deficiency and GH resistance. This novel double‐knockout model has similar physiological and metabolic features, which are associated with healthy aging and extension of lifespan, to other GH‐related long‐lived mutant mice. Finally, and most importantly, our preliminary results from longevity study suggest that D‐KO mice have extended lifespan. We believe our model will help better understand the interplay between GH deficiency and GH resistance in the context of healthspan and lifespan.

## 2 RESULTS

**FIGURE 1:**
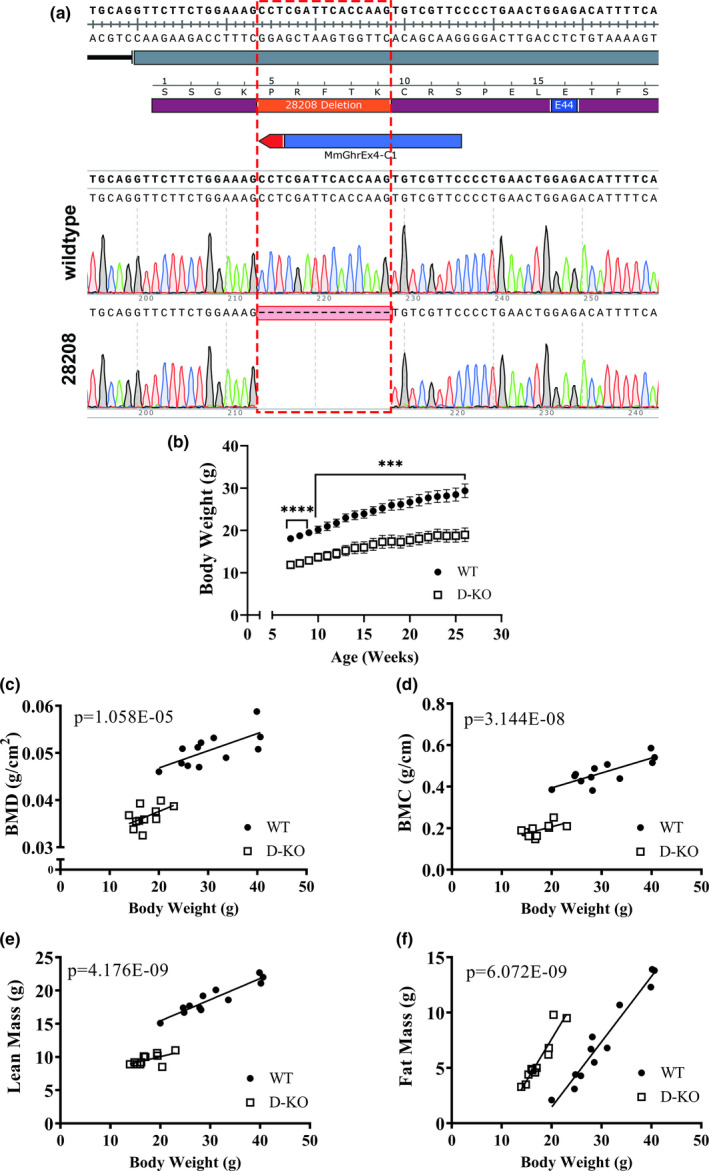
GHR/GHBP knockout mice with CRISPR technology have altered body composition. The alignment of exon 4 (gray rectangle) reference sequence with electropherograms from wild‐type and mutant (28208) alleles (a). The translated sequence of single letter amino acids (maroon rectangle) shows the five amino acids inferred to be deleted in the mutant allele (orange rectangle) and position of a missense mutation at residue E44 (blue rectangle) in a young human patient. The CRISPR target site is indicated by the blue bar, with a red arrowhead representing the PAM (NGG). The red rectangle with dotted lines in the bottom electropherogram indicates the 15 bp deleted sequence. Body weights of WT and D‐KO mice from weaning to adulthood (b). Body composition parameters were measured by DXA. Body composition parameters were plotted on the y‐axis, and body weights are plotted on the x‐axis (c‐f). Relationship between body weight and BMD in WT and D‐KO mice (c). Relationship between body weight and BMC in WT and GHRH^‐/‐^ mice (d). Relationship between body weight and lean mass in WT and D‐KO mice (e). Relationship between body weight and fat mass in WT and GHRH^‐/‐^ mice (f). WT n = 12, D‐KO n = 11. The WT and D‐KO groups were statistically analyzed with ANCOVA method, which was used to calculate p values, shown on each panel

We generated homozygous GHR knockout mice utilizing CRISPR/Cas9‐mediated gene‐editing method. In a litter of 10 G_0_ pups, 2 carried deletions in the GHR locus. One allele (#28208), a 15 base‐pair deletion that eliminates a portion of the exon 4, was identified. Predicted translation of the mutant sequence shows an in‐frame mutation resulting in the loss of 5 amino acids (PRFTK) located in the mature peptide (Figure 1a). The line carrying this mutant allele was crossed with GHRH^−/−^ line in order to create D‐KO animals used this study. Littermates of D‐KO mice, which are wild‐type for both GHRH and GHR, were used as controls.

To confirm the effect of GH‐related mutations on the expression of GH, we measured GH gene expression in pituitary tissue (Figure S1A). D‐KO group had dramatically decreased GH expression, while GHR‐KO group had increased GH expression. We found that GH expression was very similar between GHRH‐KO and D‐KO groups (Icyuz et al., 2020). We were not able to detect GHRH expression in hypothalamus by real‐time qPCR analysis in neither GHRH‐KO, nor D‐KO lines (Figure S1B). GHR‐KO group showed in slight, but significant increase in GHRH expression.

Dramatic decrease in body size and altered body composition parameters are well‐documented physiological features among GH‐related mutant mice. Longitudinal measurements of body weight in mice, from 7 to 26 weeks of age, show D‐KO mice were significantly lighter than their WT littermates (Figure 1b). We performed dual‐energy X‐ray absorptiometry (DXA) to study the body composition parameters in our novel D‐KO mice. DXA measurements found that D‐KO had significantly decreased absolute bone mineral density, bone mineral content, and lean mass compared to control littermates (Figure S2A‐C). Absolute fat mass was slightly, but not significantly, lower in D‐KO mice (Figure S2D). In order to adjust for the significant differences in body weight between mutant and normal mice, we used analysis of covariance (ANCOVA) method, which showed that BMD, BMC, and lean mass were reduced in D‐KO mice (Figure 1c‐e). However, body weight‐adjusted fat mass was significantly elevated in D‐KO mice compared to littermate controls (Figure 1f).

Body weight was dramatically decreased in GHRH‐KO, GHR‐KO, and D‐KO mice compared to WT controls. GHRH‐KO mice were slightly, but not significantly heavier than GHR‐KO and D‐KO mice (Table 1). Next, we sought to investigate whether the serum biochemical parameters were changed in D‐KO mice (Table 1). In D‐KO mice, serum IGF‐1 levels were decreased by more than 90%. This decrease was smaller in GHR‐KO mice, around 85%, and we observed least amount of decrease in GHRH‐KO mice, around 50% (Table 1). All three mutant groups had significantly decreased circulating IGF‐1 compared to WT controls. In addition, IGF‐1 levels were significantly different among GHRH‐KO, GHR‐KO, and D‐KO groups. Circulating leptin levels were increased in all three mutant groups compared to the WT controls. We observed statistical significance between the three mutant groups, where GHRH‐KO had the highest and GHR‐KO had the lowest leptin levels (Table 1). High‐molecular‐weight (HMW) adiponectin levels in serum were dramatically increased in GHRH‐KO, GHR‐KO, and D‐KO groups. GHR‐KO groups had the highest and D‐KO group had the lowest HMW adiponectin levels among the three mutant groups, all of which had significantly different levels (Table 1). Serum somatostatin levels were lower in GHRH‐KO mice and higher in GHR‐KO mice compared to WT controls. D‐KO and WT mice had very similar somatostatin levels (Table 1).

**TABLE 1:** Physiological parameters changed in D‐KO mice

	WT	GHRH‐KO	GHR‐KO	D‐KO
Body weight (g)	31.65 ± 1.86^a^	20.15 ± 0.53^b^	17.15 ± 0.72^b^	17.48 ± 0.83^b^
IGF−1 (ng/ml)	66.98 ± 1.48^a^	31.54 ± 0.72^b^	9.42 ± 0.23^c^	5.77 ± 0.59^d^
Leptin (ng/ml)	8.36 ± 0.21^a^	15.08 ± 0.66^b^	9.81 ± 0.47^c^	13.32 ± 0.24^d^
HMW adiponectin (μg/ml)	12.19 ± 0.70^a^	21.95 ± 0.47^b^	23.3 ± 0.03^c^	18.39 ± 0.67^d^
Somatostatin (ng/ml)	1.02 ± 0.04^a^	0.79 ± 0.03^b^	1.3 ± 0.09^c^	0.97 ± 0.08^ab^

Note: The 4‐hour fasting mice were used to collect the serum. The various serum parameters were analyzed in WT, GHRH‐KO, GHR‐KO, and D‐KO mice. Data represent the means ±SEM. n = 14–18 per genotype for body weight, n = 6–8 mice per genotype for blood parameters. Different superscripts denote significant difference at *p* < 0.05.

**FIGURE 2:**
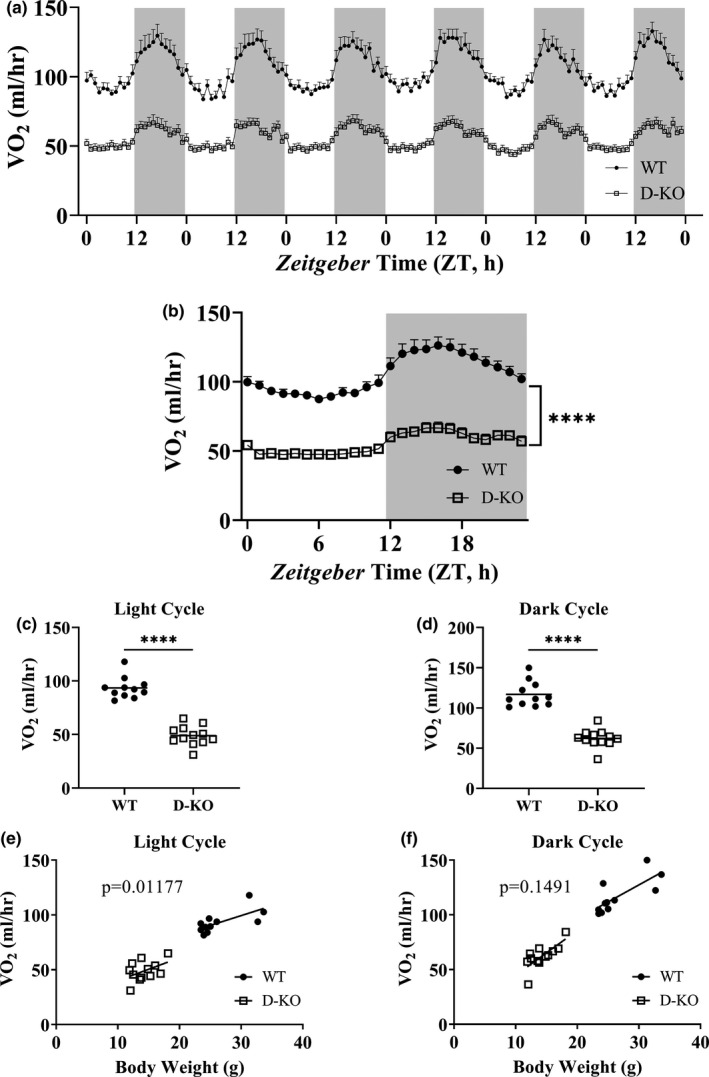
VO_2_ recordings in D‐KO mice. VO_2_ was measured by indirect calorimetry for 6 days in WT and D‐KO mice (a). 6 days of VO_2_ data were averaged into a single day format (b). VO_2_ recordings measured on light (c) and dark (d) cycles were averaged for individual animals. VO_2_ is plotted on the y‐axis, and body weights are plotted on the x‐axis for light (e) and dark (f) cycles. WT n = 11, D‐KO n = 12. Each bar represents mean ±SEM. For panels c‐d, statistical analysis was performed with unpaired Student's t test with Welch's correction; ****p < 0.0001. For panels e and f, statistical significance was determined by ANCOVA

**FIGURE 3:**
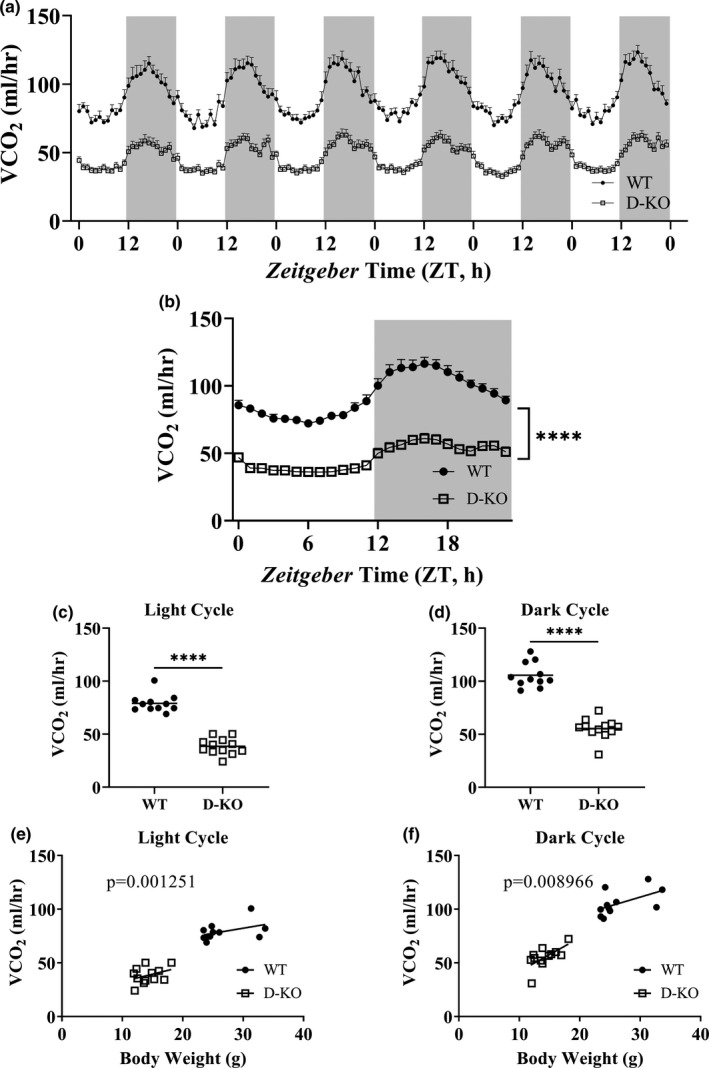
VCO_2_ recordings in D‐KO mice. VCO_2_ was measured by indirect calorimetry for 6 days in WT and D‐KO mice (a). 6 days of VCO_2_ data were averaged into a single day format (b). VCO_2_ recordings measured on light (c) and dark (d) cycles were averaged for individual animals. VCO_2_ is plotted on the y‐axis, and body weights are plotted on the x‐axis for light (e) and dark (f) cycles. WT n = 11, D‐KO n = 12. Each bar represents mean ±SEM. For panels b‐d, statistical analysis was performed with unpaired Student's t test with Welch's correction; ****p < 0.0001. For panels e and f, statistical significance was determined by ANCOVA

To assess the influence of altered GH signaling on respiratory parameters, we performed indirect calorimetry by recording oxygen consumption (VO_2_) and carbon dioxide production (VCO_2_) for 6 days in D‐KO mice (Figures 2a and 3a). VO_2_ and VCO_2_ measurements collected for 6 days were averaged into a single day, further highlighting the significantly lower respiration rates in D‐KO mice (Figures 2b and 3b). Overall averages of absolute VO_2_ and VCO_2_ measurements were significantly lower in D‐KO mice compared to WT littermates in both light and dark cycles (Figures 2c‐d and 3c‐d). However, body weight‐adjusted VO_2_ of D‐KO was decreased only during the light cycle (*p* = 0.01177) but not the dark cycle (Figure 2e, f). Body weight‐adjusted VCO_2_ of D‐KO was lower during both light (*p* = 0.001251) and dark (*p* = 0.008966) cycles (Figure 3e, f).

**FIGURE 4:**
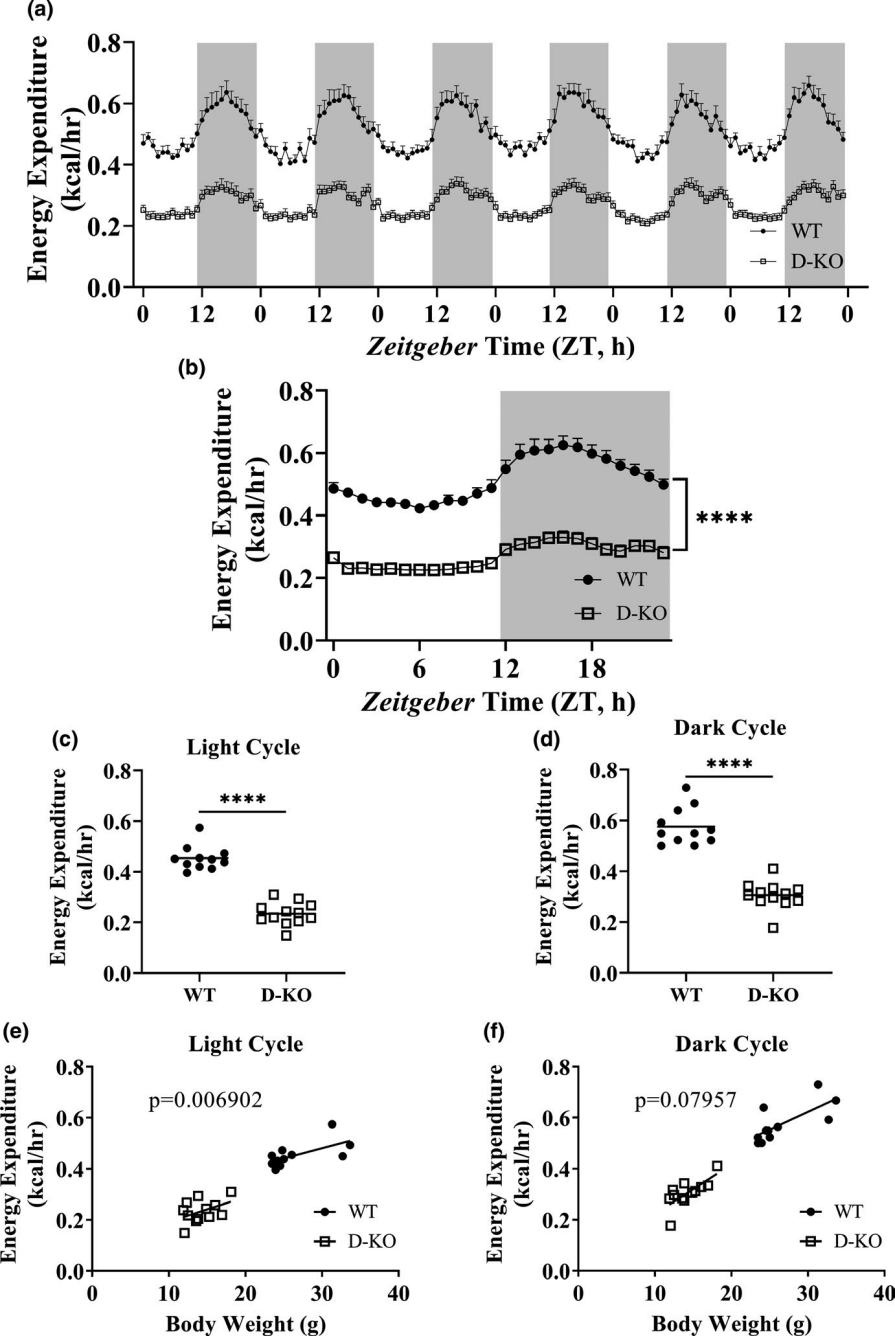
Metabolic rate in D‐KO mice. Energy expenditure was calculated using respiratory parameters measure by indirect calorimetry for 6 days in WT and D‐KO mice (a). 6 days of energy expenditure data were averaged into a single day format (b). Energy expenditure values on light (c) and dark (d) cycles were averaged for individual animals. Energy expenditure values are plotted on the y‐axis, and body weights are plotted on the x‐axis for light (e) and dark (f) cycles. WT n = 11, D‐KO n = 12. Each bar represents mean ±SEM. For panels b‐d, statistical analysis was performed by unpaired Student's t test with Welch's correction; ****p < 0.0001. For panels e and f, statistical significance was determined by ANCOVA

To assess the effect of GH resistance/deficiency on metabolic rate, we calculated energy expenditure using the data collected by indirect calorimetry. Absolute energy expenditure was lower throughout 6 days in D‐KO mice compared to WT littermates (Figure 4a). Figure 4b presents absolute energy expenditure data collected for 6 days averaged into a single day format. Statistical analyses of absolute energy expenditure levels indicated a significant decrease in D‐KO mice in both light and dark cycles (Figure 4c, d). We adopted ANCOVA method to remove the effect of differences of body weight between D‐KO and WT littermates on energy expenditure. This analysis suggested that D‐KO mice have significantly lower energy expenditure during the light (*p* = 0.006902) but not the dark cycle (*p* = 0.07957) (Figure 4e, f).

**FIGURE 5:**
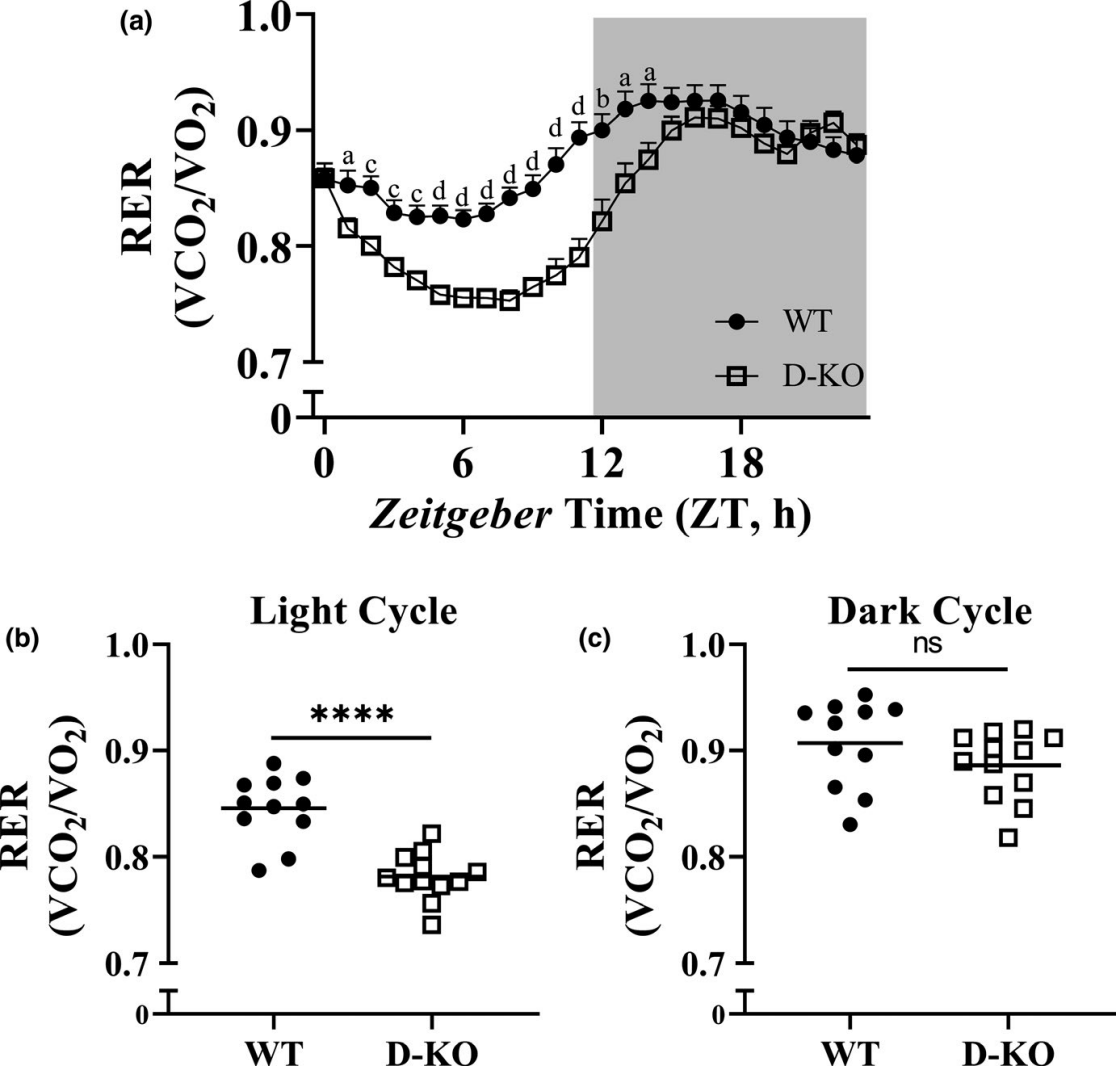
Respiratory exchange ratio (VCO_2_/VO_2_) in D‐KO mice. RER values were calculated by dividing VCO_2_ with VO_2_. RER data, which were collected during a 6‐day period, were averaged into a single day format (a). Averaged RER values are shown as light (b) and dark (c) cycles for WT and D‐KO mice. Female WT n = 11, D‐KO n = 12. Each bar represents mean ±SEM. Statistical analysis was performed with unpaired Student's t test with Welch's correction; ns=not significant, a; *p < 0.05, b; **p < 0.01, c; ***p < 0.001, d; ****p < 0.0001

To identify substrate utilization profile of D‐KO mice, we calculated RER by dividing VCO_2_ with VO_2_. RER values averaged into single day format support that throughout the light cycle, and during the early dark cycle hours D‐KO mice have significantly lower RER compared to WT littermates (Figure 5a). The analyses of RER collected from 6 light and dark cycles provide further evidence for the remarkably altered metabolism in circadian manner in D‐KO mice (Figure 5b, c). During indirect calorimetry recordings, we measured voluntary physical activity. D‐KO mice had significantly less ambulatory activity during both the light and dark cycles (Figure S3B, E, F). However, locomotor activity was only reduced during the light cycle for D‐KO mice compared to WT littermates (Figure S3A, C, D).

**FIGURE 6:**
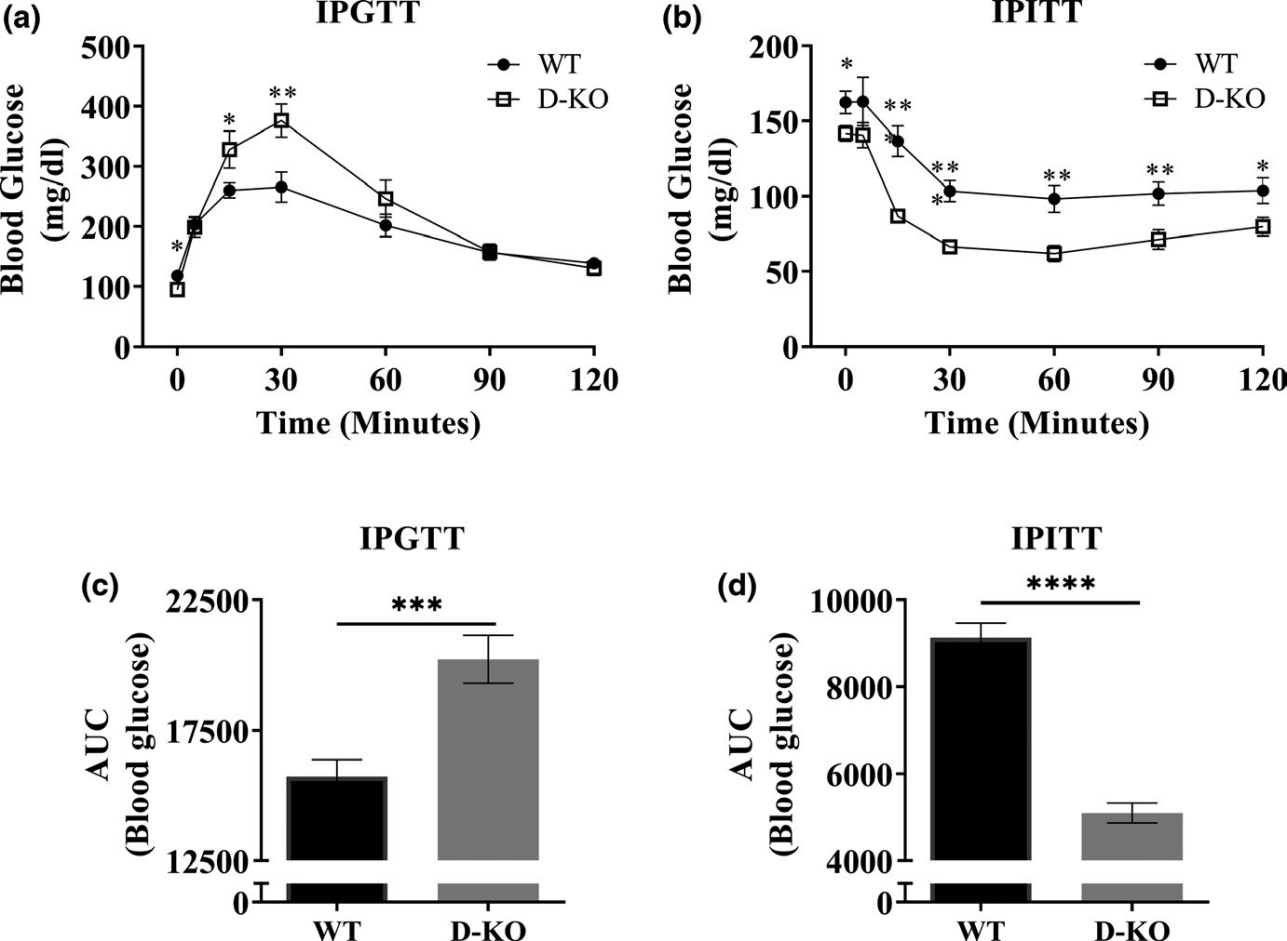
Insulin and glucose tolerance tests. D‐KO and WT mice were fasted overnight and injected with 1 g glucose per kg of body weight. Blood glucose levels of mice were measured during the following 2 hours (a). Area under the curve analysis is shown (c). D‐KO and WT mice were fasted for 4 hours and injected with 1 IU porcine insulin per kg of body weight. Blood glucose levels of mice were measured during the following 2 hours (b). Area under the curve analysis is shown (d). WT n = 13–14, D‐KO n = 10–11. Each bar represents mean ±SEM. Statistical analysis was performed with unpaired Student's t test with Welch's correction; *p < 0.05, **p < 0.01, ***p < 0.001, ****p < 0.0001

We performed intraperitoneal glucose tolerance tests (IPGTT) to evaluate glucose homeostasis in D‐KO mice. Overnight‐fasted D‐KO had higher resting blood glucose levels compared WT littermates (Figure 6a). However, at the 15‐ and 30‐minute time points blood glucose levels were much higher in D‐KO mice (Figure 6a). This difference subsided at 1 hour, and blood glucose levels of D‐KO and WT mice remained very similar during the 2^nd^ hour (Figure 6a). Area under the curve of blood glucose levels throughout the IPGTT was significantly higher for D‐KO mice compared to WT littermates (Figure 6c). Next, we performed intraperitoneal insulin tolerance tests (IPITT). After fasting mice for 4 hours, blood glucose levels were higher in D‐KO mice compared to WT littermates (Figure 6b). We observed dramatically lower blood glucose levels in D‐KO mice throughout the 2‐hour period following the insulin injection, except for the 5‐minute time point (Figure 6b). Area under the curve of blood glucose levels throughout the IPITT was dramatically lower for D‐KO mice compared to WT littermates (Figure 6d).

Currently, we are 2 years into a longevity study with D‐KO mice. We are executing this study with 29 WT and 24 D‐KO mice. So far, we have recorded 13 WT mice deaths but 2 deaths in the D‐KO cohort (Figure S4).

## 3 DISCUSSION

The positive impact of GH signaling on longevity has been well documented by genetically targeting different components of this pathway, which plays an important role in the development of many tissues in the body. Previously, a double‐mutant mouse line was generated by crossing GHR/GHBP‐KO mice with Ames dwarfs (Gesing et al., 2017), which are deficient in prolactin and thyroid‐stimulating hormone in addition to GH deficiency (Heiman et al., 2003). In order to elucidate the role of GH signaling without being confounded by other hormone deficiencies, we generated this isolated D‐KO mouse model via CRISPR/Cas9 technology. We recently reported the novel CRISPR/Cas9 GHRH‐KO mice (Icyuz et al., 2020). In this study, we employed the same strategy to disrupt GHR/GHBP gene and bred the resulting mouse with GHRH‐KO to generate a novel D‐KO mouse model for GH signaling. Unfortunately, we were not able to produce enough D‐KO males to conduct our studies. It is probable that this skewed sex ratio might be due to the disruption of the two critical components GH signaling simultaneously.

The targeted editing of GHR/GHBP gene resulted in deletion of 5 amino acids residing close to the E44 residue, which was shown to be in direct contact with GH according to the crystal structure of the complex between GH and its receptor (Figure 1a) (de Vos et al., 1992). Furthermore, replacement of E44 with alanine was shown to reduce binding to GH (Clackson & Wells, 1995). With the loss of the 5 amino acids in close proximity to this critical residue (E44), the binding between the extracellular domain of GHR and GH is predicted to be negatively affected to a great extent leading to downregulation of further downstream GH signaling. This is likely to give rise to the loss‐of‐function phenotype.

Decreased body weight is one of the most common phenotypes of diminished growth hormone signaling in mouse models, including Ames, Snell, GHRH‐KO, and GH receptor protein/GH‐binding protein‐deficient mice. Our novel D‐KO mouse model presents a dramatically lower body weight from 7 weeks of age to 26 weeks of age. Our findings further highlight the numerous observations in aging studies, which show body weight is inversely related to longevity. When we compared the body weights our three GH‐related KO models, we did not find significant differences between them.

To elucidate the mechanistic influence of different GH‐related mutations on longevity, we examined serum levels of hormones, which have been strongly associated with extended longevity in these GH‐related mutant mice. Reduced circulating IGF‐1 levels are a hallmark of longevity among GH‐resistant and GH‐deficient mouse models (Chandrashekar & Bartke, 1993; Sun et al., 2013; Zhou et al., 1997). Similar to previous studies, GHRH‐KO and GHR‐KO groups had lower IGF‐1 compared to control group (Sun et al., 2013; Zhou et al., 1997). D‐KO mice had the lowest and GHRH‐KO mice had the highest serum IGF‐1 levels among the three mutant lines. Studies suggest that adiponectin plays a critical role in regulation of glucose and lipid metabolism and decreased circulating adiponectin levels have been implicated in the development of insulin resistance in mammalian models (Yamauchi et al., 2001). In this study, we measured HMW adiponectin, which is thought to be the most active form of adiponectin, mediates insulin sensitization (Wang et al., 2008). Compared to the healthy human population, HMW adiponectin levels are significantly increased in patients with molecular defects in GHR gene (Laron syndrome) (Kanety et al., 2009). Similarly, GHR‐KO mouse model of the Laron syndrome showed increased levels of HMW adiponectin (Lubbers et al., 2013). HMW adiponectin levels were found to be significantly elevated in all three mutant lines relative to the control group, and GHR‐KO mice had the highest. The D‐KO mice had the lowest HMW adiponectin among the three mutant lines. Leptin is increased in mice with GH‐related mutations (Al‐Regaiey et al., 2005; Sun et al., 2013). In addition, overexpression of leptin was shown to rescue insulin resistance in diabetic mouse models (Ebihara et al., 2001). In the present study, all three KO lines had significantly increased circulating leptin with GHRH‐KO line having the highest and GHR‐KO line the lowest levels. We found that circulating somatostatin levels in GHR‐KO mice was higher than in WT controls. In addition, we demonstrated increased GH expression in pituitary of GHR‐KO mice. These results are delineating a well‐known negative feedback loop, in which high levels of circulating growth hormone increase the release of somatostatin, thereby lowering secretion of growth hormone. The same feedback loop in GHRH‐KO mice may have contributed to these findings which had increased circulating somatostatin and decreased GH expression in pituitary. However, D‐KO mice had very similar circulating somatostatin levels to WT controls. This result suggests that absence of both GHRH and GHR might be canceling out each other's effect on the release of somatostatin.

Overall, the critical differences in IGF‐1, HMW adiponectin, and leptin between the three KO lines suggest that GH signaling pathway is more complex that initially thought be. In the context of the D‐KO, the combinatorial effect of GHRH and GHR KOs appears to be different than the sum of individual mutation's effects (Mani, St Onge, Hartman, Giaever, & Roth, 2008).

Our study relies heavily on accurate understanding of physiological and metabolic variables, which are dependent on body weight. Therefore, it is crucial to use the most accurate model of statistical analysis for valid interpretation of results. We are avoiding reporting physiological variables normalized with ratio‐based methodology, which has been demonstrated to produce misleading conclusions (Tanner, 1949). We present body composition and metabolic parameters as collected from individual animals and in addition, we used ANCOVA, which accounts for differences in body weight between different groups of animals and is the most widely accepted statistical model in this scenario (Kaiyala & Schwartz, 2011; Tschop et al., 2011).

Significantly lower absolute BMD, BMC, and lean mass were observed in novel D‐KO model similar to the GHRH‐KO model in our previous study. In addition, analyses of body composition parameters corrected for body weight showed that BMD, BMC, lean mass, and fat mass were significantly lower in D‐KO mice. In our recent study with the GHRH‐KO model, we detailed indirect calorimetry protocol, which was intended to improve reproducibility and interpretation of the resulting data from different studies, and was applied to our current study. We observed dramatic decreases in absolute VO_2_ and VCO_2_ in D‐KO mice, during light and dark cycles. VO_2_ and VCO_2_ measurements corrected for body weight with ANCOVA method showed a similar pattern except for the VO_2_ recorded during the dark cycle, which was not significantly different between D‐KO and WT mice. Absolute metabolic rate during both light and dark cycles was significantly lower in D‐KO mice. Body weight corrected energy expenditure was significantly lower in D‐KO mice during the light cycle, but not during the dark cycle.

RER is deduced by dividing VCO2 by VO2. A RER close to 0.7 indicates metabolism lead by utilizing fats, whereas a RER close to 1.0 indicates metabolism dominated by carbohydrate reduction. Results show decreased RER during the light cycle in the D‐KO, which indicates increased fat metabolism during rest compared to WT littermates. This trend is followed early in the dark cycle when averaging the 7 days, demonstrating D‐KO utilizes fat metabolism longer than WT littermates.

Differences in physical activity exist in the D‐KO model. Locomotor activity and ambulatory activity are both decreased during normally active times for mice. A similar study was done on locomotor activity with GHRH‐deficient mice; however, data were only collected for 10 minutes during this study (Leone et al., 2018). Along with the delay in carbohydrate metabolism, these results may illustrate that the D‐KO mice are at rest longer than WT littermates.

Insulin sensitivity is a remarkably consistent characteristic of mouse model, which have increased longevity and healthspan, including our novel D‐KO model. This is expected as both GH‐resistant and GH‐deficient models are insulin sensitive. However, glucose tolerance is rather inconsistent among mouse models of longevity. D‐KO mice are glucose intolerant similar to GHR‐KO mice, whereas they are dissimilar to GHRH‐KO mice and many other models with increased insulin sensitivity (Holzenberger et al., 2003; Junnila et al., 2016; Selman & Withers, 2011; Sun et al., 2013; Taguchi et al., 2007). These findings suggest the dominant role of GH resistance over GH deficiency on glucose tolerance. This non‐intuitive glucose intolerance with insulin sensitivity may be due to low β‐cell mass, leading to impaired effects of glucose clearance, which is also seen in GHR knockout models (Guo et al., 2005).

Finally, our ongoing lifespan study, which started approximately 2 years ago, indicates that D‐KO mice have increased longevity compared to WT controls. It is interesting to point out that a recent study by Gesing et al. evaluated the lifespan of the double‐mutant (df/KO) mice by crossing GHR‐KO mice with Ames dwarf mice (Gesing et al., 2017). Intriguingly, the (df/KO) mice did not exhibit any further longevity advantage when compared with Ames dwarf or GHR‐KO mice. Other physiological markers and molecular characterization show the similar pattern between df/KO mice and Ames dwarf/GHR‐KO mice. Their results indicate that either GH receptor disruption or GH deficiency by prop‐1 mutation might be sufficient to achieve the maximal longevity benefit. Unfortunately, our current study is not able to make the similar comparison. Thus, it will be critical in the future experiments to address this important issue using our novel D‐KO model.

## 4 EXPERIMENTAL PROCEDURES

### 4.1 CRISPR/sgRNA design and synthesis

Using the CRISPR design tool in Benchling (www.benchling.com), we aimed at identifying a high scoring (low off‐target) site in one of the early exons so as to generate indels that can create a loss‐of‐function allele; we found a target site in exon 4. Single guide RNA (sgRNA) molecules were generated using a cloning‐free method as described earlier (Turner et al., 2018). Cas9 protein was obtained from MacroLabs at UC Berkeley.

### 4.2 Generation of G0 (founder) animals and germline transmission of mutant alleles

All animal procedures were performed in accordance with the recommendations in the guide for the care and use of laboratory animals published by the National Institutes of Health. The protocols used were approved and conducted according to the University of Alabama at Birmingham Institutional Animal Care and Use Committee (UAB‐IACUC). Pronuclear injections into C57BL/6 J zygotes were performed with a solution of sgRNAs (50 ng/μl each) and Cas9 protein (50 ng/μl per guide). Injected zygotes were implanted into pseudo‐pregnant CD1 recipients. Genomic DNA obtained from tail biopsies of putative founder (G_0_) animals was assessed for the presence of mutations in the targeted genes. G_0_ animals were bred to WT C57BL/6 J mice for germline transmission of mutant alleles. Our colony of D‐KO mice of produced litters that are skewed toward females. We are not able to produce enough male D‐KO mice to conduct our studies. Therefore, only female mice were used in the current study.

### 4.3 Detecting the presence of indels in the mutant animals

A 478 bp PCR amplicon having the CRISPR target site was generated by using the single stranded oligonucleotide primers GhrEx4‐gen‐F1‐5’‐CCAGAGAGACTGGCTTTATCTTC‐3’ and GhrEx4‐gen‐R1‐5’‐CTCCAAAGCCTCTCCATCATATAC‐3’, and subjected to heteroduplex mobility assay (HMA) using TBE‐polyacrylamide gel electrophoresis.

### 4.4 Dual‐energy X‐ray absorptiometry (DXA)

We performed DXA scans with the GE Lunar PIXImus DXA with software version 1.45. We delivered isoflurane (3%) and oxygen (500 ml/min) mixture by a Surgivet anesthesia machine to anesthetize the mice. Mice were placed in a prostrate position on the DXA imaging plate. The heads of the mice were excluded from the examination of BMC, BMC, lean mass, and fat mass.

### 4.5 Serum collection and ELISA

The mice were fasting 4 hours before collection of the whole blood. The serum was collected by centrifuge at 2,000 x g for 10 minutes at 4°C. The ELISA was preformed according to the manual. The leptin ELISA kit (catalog # 90030) was purchased in Crystal Chem, IGF‐1 ELISA kit (catalog # MG100) was R&D SYSTEM, the high‐molecular‐weight adiponectin ELISA kit (catalog # 47‐ADPMS‐E01) was ALPCO, and somatostatin ELISA kit (EK‐060–03) was phoenix pharmaceuticals.

### 4.6 Indirect calorimetry

We conducted indirect calorimetry using comprehensive laboratory animal monitoring system (Oxymax‐CLAMS; Columbus Instruments Co., Columbus, OH). This system uses zirconia and infrared sensors to monitor oxygen (O_2_) and carbon dioxide (CO_2_), respectively. We housed mice in individual respiratory chambers for 7 days in order to acclimate mice to single housing conditions. Oxygen consumption and carbon dioxide production parameters were recorded for 6 days with ad libitum access to standard chow and water. Respiratory samples were measured every 9 minutes for each animal, and the data were averaged for each hour. RER was calculated by dividing VCO_2_ by VO_2_. Energy expenditure was calculated by the equation as energy expenditure = (3.815 + 1.232 × VCO_2_/VO_2_) × VO_2_ (Icyuz et al., 2020). We used infrared beam system in X, Y, and Z coordinates to record physical activity of mice.

### 4.7 Glucose and insulin tolerance tests

Overnight‐fasted mice underwent glucose tolerance test by intraperitoneal injection with 1 g of glucose per kg of body weight. 4‐hour fasted mice underwent insulin tolerance test by intraperitoneal injection with 1 IU porcine insulin (Sigma‐Aldrich, St. Louis, MO) per kg of body weight. Blood glucose levels were measured by a PRESTO glucometer using tail vein blood at 0, 5, 15, 30, 60, 90, and 120 minutes.

### 4.8 Real‐time quantitative PCR

RNA was harvested from tissues using RNeasy plus kit (Qiagen, Hilden, Germany). Total RNA was reverse‐transcribed with LunaScript RT SuperMix Kit (New England Biolabs, Ipswich, MA). Real‐time quantitative PCR was performed using a QuantStudio 3 with a PowerUp SYBR Green Master Mix (Thermo Fisher Scientific, Waltham, MA). Glyceraldehyde‐3‐phosphate dehydrogenase (GAPDH) or beta‐actin expression was used to normalize gene of interest in each sample. Real‐time quantitative PCRs were set up using the oligonucleotide primers Mm GAPDH F1 5‐CCTGGAGAAACCTGCCAAGTATGATG‐3’; Mm GAPDH R1 5‐AAGAGTGGGAGTTGCTGTTGAAGTC‐3’, Mm Actb F4 5’‐TCTTTGCAGCTCCTTCGTTGCC‐3; Mm Actb R4 5’‐CTGACCCATTCCCACCATCACAC‐3’, Mm GHRH F1 5’‐GGTGCTCTTTGTGATCCTCATC‐3’; Mm GHRH R1 5’‐GTTTCCTGTAGTTGGTGGTGAAG‐3’; Mm GH F1 5’‐ TGGCTACAGACTCTCGG‐3’; Mm GH R1 5’‐AGAGCAGGCAGAGCAGGCTGA‐3’. Fold change was obtained by calculating 2^−∆∆Ct^.

### 4.9 Statistical analyses

The unpaired Student's t test with Welch's correction and one‐way ANOVA with Benjamini and Hochberg false discovery rate (FDR) were used for statistical analysis. Statistical significance was established at *p* < 0.05, two‐tailed. For analyses of indirect calorimetry and body composition data, we used (generalized linear model) GLM package with R software. Graphs were generated with GraphPad Prism 8 (San Diego, CA).

## CONFLICTS OF INTEREST

All of the contributing authors declared no conflicts of interest.

## AUTHOR CONTRIBUTIONS

Liou Y. Sun conceived the study and was in charge of overall direction and planning. Michael P. Fitch, Mert Icyuz, Fang Zhang, and Matthew R. Joyner performed experiments and collected data. Anil K. Challa performed CRISPR experiments. Mert Icyuz took the lead in writing the manuscript. All authors provided critical feedback and helped shape the research, analysis, and manuscript.

## Supporting information

Fig S1‐S4

## Data Availability

The data that support the findings of this study are available in the supplementary material of this article.
