# Supplementary material for: Physiological and metabolic characteristics of novel double‐mutant female mice with targeted disruption of both growth hormone‐releasing hormone and growth hormone receptor
Source: Aging Cell. 2021 Mar 23;20(4):e13339. doi: 10.1111/acel.13339 (PMC8045953; doi:10.1111/acel.13339)

Supplemental Figure Legends

Supplemental Figure 1. D-KO mice have increased GH and GHRH expression.

Expression of growth hormone gene was measured in pituitary gland in GHRH-KO, GHR-KO, D-KO and WT mice. Expression of growth hormone releasing hormone gene was measured in hypothalamus in GHRH-KO, GHR-KO, D-KO and WT mice. For all biological groups n=8. Each bar represents means. Statistical analysis was performed with one-way ANOVA with Benjamini and Hochberg false discovery rate (FDR); different letters denote significant difference at P < 0.05.

Supplemental Figure 2. D-KO mice have altered body composition parameters.

*Body composition parameters: BMD (A), BMC (B), lean mass (C) and fat mass (D) were measured by DXA. WT n=12, D-KO n=11. Each bar represents mean ± SEM. Statistical analysis was performed by unpaired Student's t-test with Welch's correction; ns= not significant, ***p<0.001, ****p<0.0001.*

Supplemental Figure 3. Physical activity in D-KO mice. *Locomotor activity (A) and ambulatory activity (B) of WT and D-KO mice during a period of 6 days are shown. WT n=11, KO n=12. Averaged locomotor activity (C, D) and ambulatory activity (E, F) are shown as light (C, E) and dark (D, F) cycles for WT and D-KO mice. Each bar represents mean ± SEM. Statistical analysis was performed with unpaired Student's t-test with Welch's correction; ns= not significant, **p<0.01, c; ***p<0.001.*

Supplemental Figure 4. Longevity of D-KO mice. *Kaplan-Meier survival curves for each genotype: D-KO (red) and WT (black) mice. WT n=28, D-KO n=24.*

Supplemental Figure 1

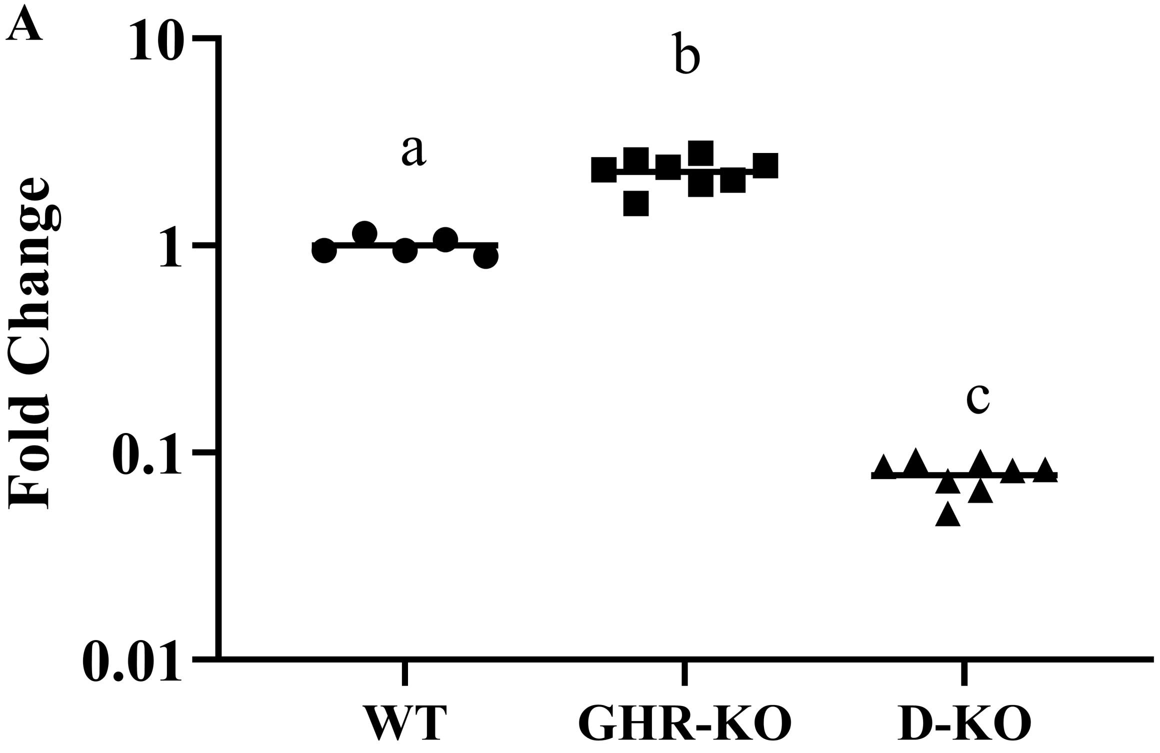

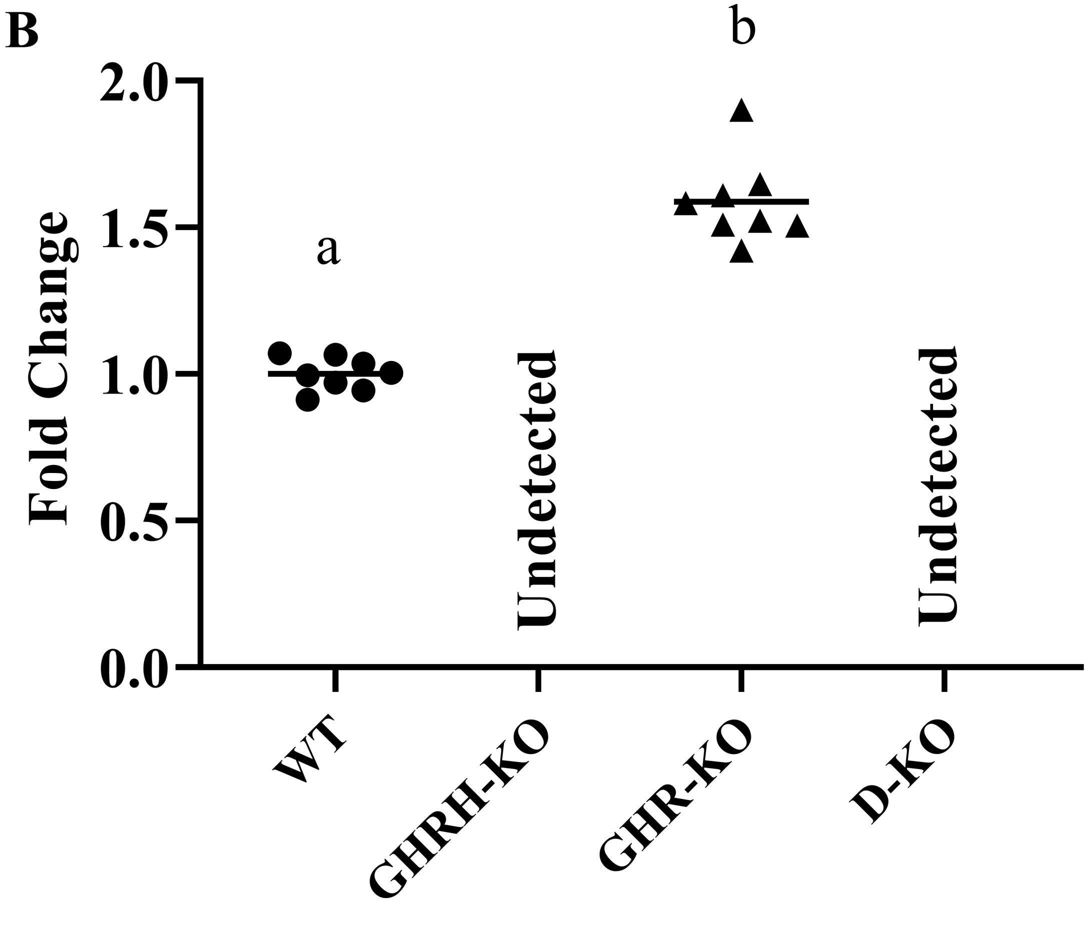

Supplemental Figure 2

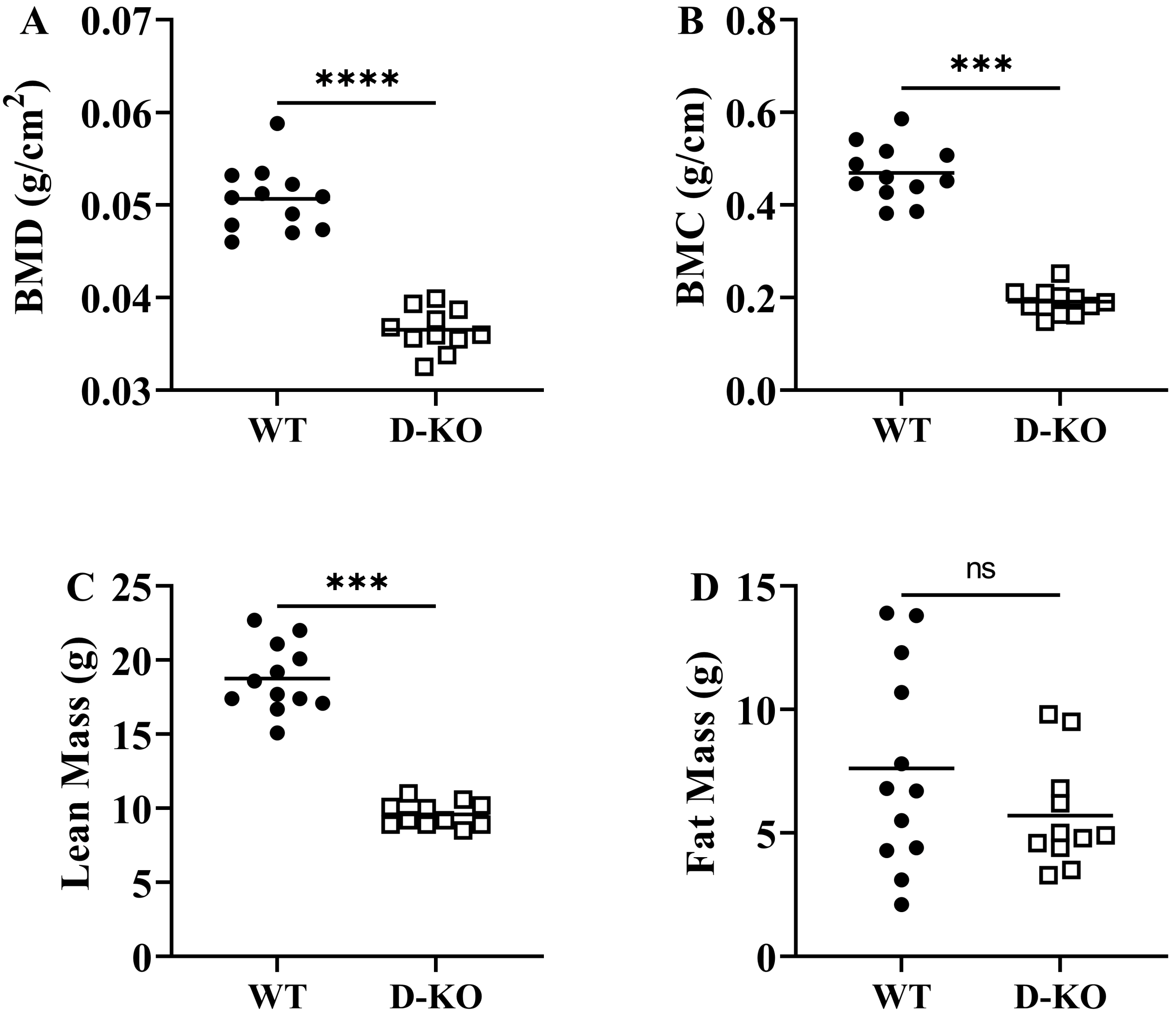

Supplemental Figure 3

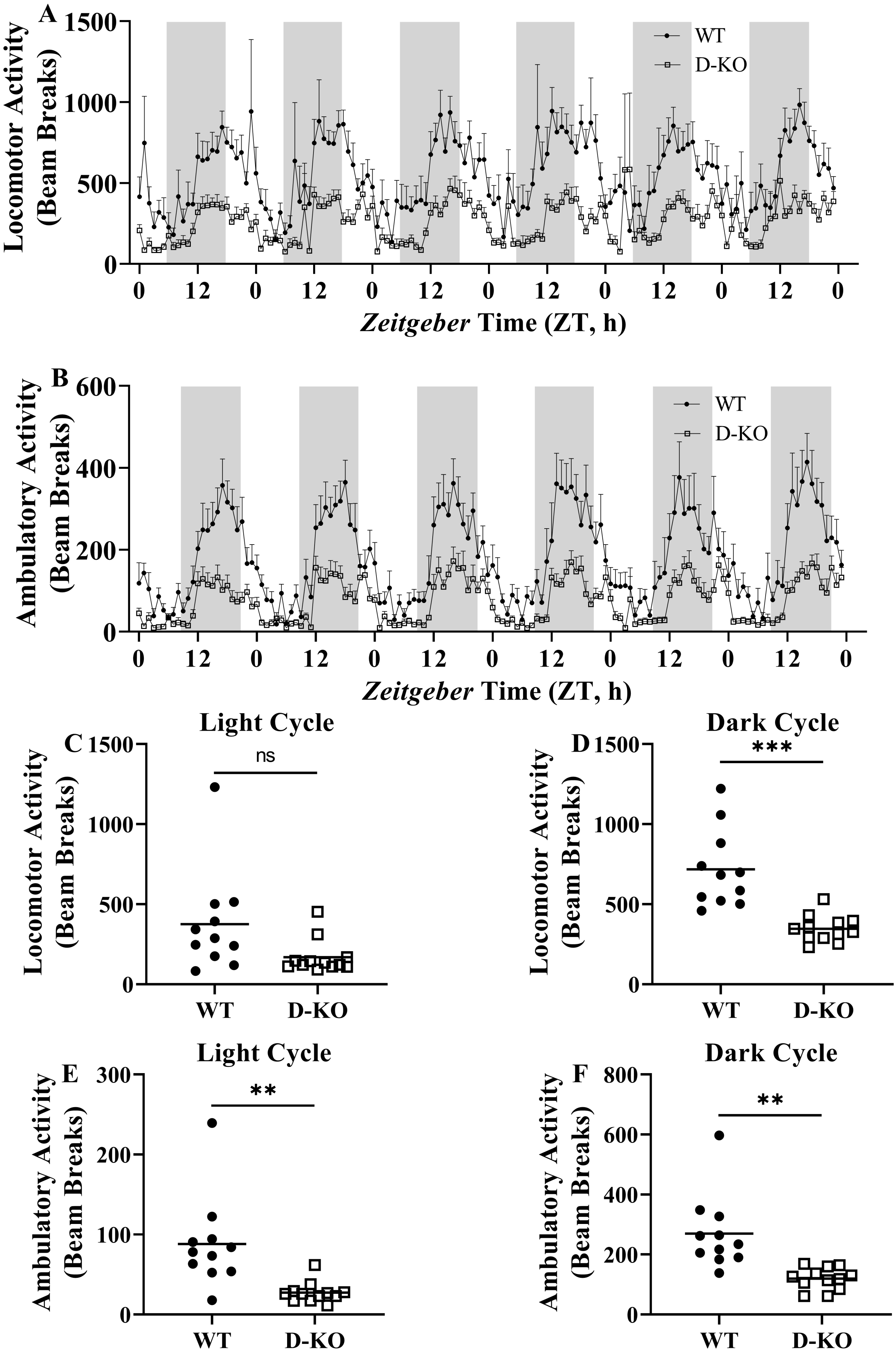

Supplemental Figure 4

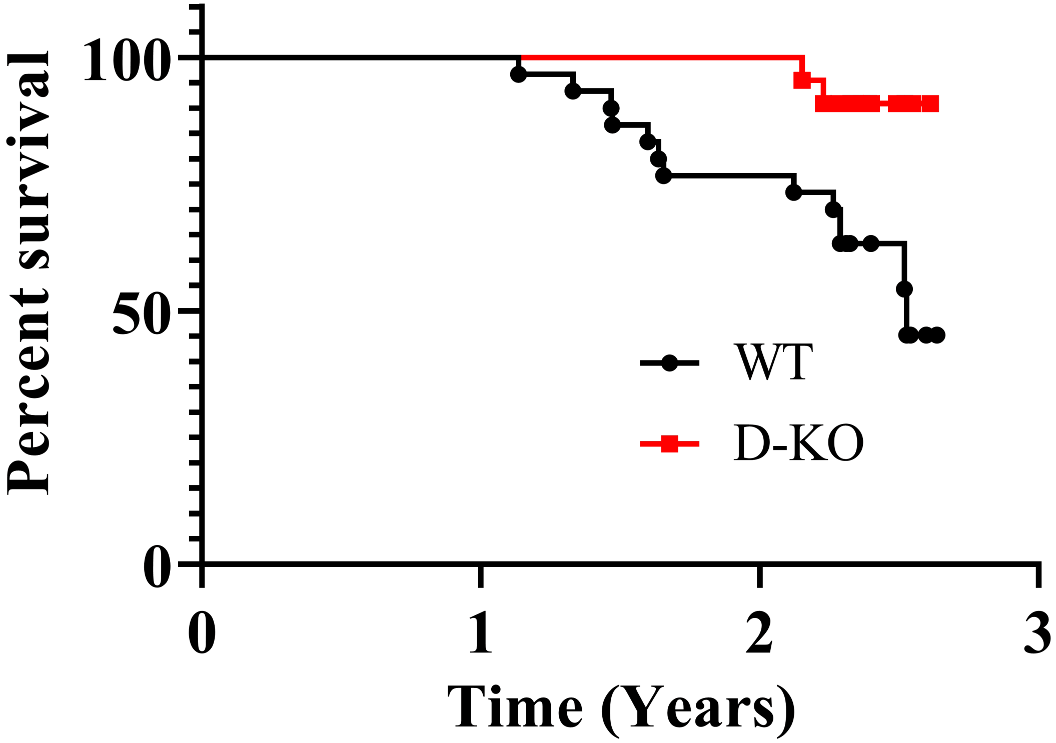

Supplement: Supplementary file 1 — Fig S1‐S4 [file ACEL-20-e13339-s001.pdf]
